# Editorial: Bioactive and Nutritional Molecules: From Natural Sources to Therapeutic and Health Applications

**DOI:** 10.3390/molecules31213824

**Published:** 2026-10-23

**Authors:** Mauro Lombardo

**Affiliations:** Department for the Promotion of Human Science and Quality of Life, San Raffaele Open University, 00166 Rome, Italy; mauro.lombardo@uniroma5.it

Natural products have historically represented one of the most important sources of biologically active molecules, providing both direct therapeutic agents and molecular scaffolds for the development of new pharmacological and nutraceutical applications [1]. In recent years, advances in analytical chemistry, molecular biology, omics technologies, and bioinformatics have substantially expanded the ability to identify, characterize, and investigate naturally derived compounds [2]. At the same time, increasing interest in prevention-oriented healthcare and in the interaction between diet, bioactive compounds, and human physiology has further strengthened the connection between natural products, nutrition, and medicine [3].

The Special Issue “Bioactive and Nutritional Molecules: From Natural Sources to Therapeutic and Health Applications” was conceived to provide a multidisciplinary overview of these rapidly evolving areas, bringing together studies addressing the isolation and characterization of natural molecules, their biological activities, mechanisms of action, and potential applications in health and disease. The nine contributions collected in this Special Issue reflect the considerable chemical and biological diversity of natural bioactive compounds, ranging from fungal polysaccharides and microbial metabolites to plant-derived phenolics, mushroom sterols, bee venom enzymes, caffeine, and functional food components. They also illustrate the broad spectrum of possible applications, including cancer, viral infections, liver health, immune modulation, hair loss, and nutritional management of dysphagia.

The first important theme emerging from the collection is the continued relevance of natural products as a source of structurally diverse bioactive molecules. Maglangit et al. [4] investigated the metabolites produced by *Streptomyces* sp. MA37, isolating the previously undescribed siderophore legonoxamine I together with streptimidone. Legonoxamine A demonstrated pronounced anticancer activity against MCF-7 breast cancer cells, outperforming desferrioxamine B—a difference that was potentially driven by its phenylacetyl moiety [4]. The structural and functional complexity of natural molecules is further illustrated by Muntean and Florea [5], who reviewed phospholipase A2 (PLA2) from *Apis mellifera* venom. Bee venom PLA2 exemplifies the dual nature of many bioactive compounds, where toxicity and pharmacological utility are intimately linked through mechanisms involving inflammation, apoptosis, and immune modulation. Evaluating such complex entities requires a comprehensive assessment of efficacy, mechanism, bioavailability, and safety [6]. The Special Issue also highlights the potential of plant-derived bioactive compounds in cancer and infectious diseases. Spagnuolo et al. [7] investigated the interaction between the natural flavonoid quercetin and mafosfamide in two human bladder cancer cell lines. Their results showed a synergistic reduction in cell viability when the compounds were combined, although the biological responses differed according to the cellular model and treatment sequence, emphasizing that natural–synthetic drug combinations depend heavily on treatment timing and cellular context. Similarly, Feng et al. [8] investigated the antiviral properties of *Moslae herba* aqueous extract against SARS-CoV-2 infection. By integrating chemical profiling, network pharmacology, transcriptomics, molecular docking, and experimental validation, the authors identified caffeic acid, luteolin, and rosmarinic acid as active antiviral constituents acting on specific targets such as PNP and TPI1. This study exemplifies the increasingly integrated approach used to connect complex natural extracts with specific molecular mechanisms, although the transition from in vitro activity to clinical efficacy remains an important challenge requiring appropriately designed pharmacokinetic, toxicological, and clinical studies [6].

The second major theme is represented by fungal-derived molecules and their potential effects on metabolic, hepatic, and immune pathways. Szelenberger and Więckowska [9] reviewed the evidence concerning fungal polysaccharides (e.g., from *Lentinula edodes*, *Grifola frondosa*, *Ganoderma lucidum*, *Coriolus versicolor*, and *Cordyceps militaris*) in liver health, paying particular attention to alcohol-related liver disease, metabolic dysfunction-associated fatty liver disease, and toxin-induced hepatic injury. These polysaccharides were associated with reductions in oxidative stress and inflammation and improvements in metabolic parameters through NF-κB, Nrf2, NLRP3, and gut microbiota-related mechanisms. These observations are particularly relevant given the growing interest in the interaction between dietary bioactive compounds, intestinal microbiota, and metabolic health [10]. This topic is complemented by the review of Chen et al. [11], which specifically examined the immunomodulatory effects of *Cordyceps* polysaccharides. Evidence from cellular and animal studies suggests that these compounds may influence macrophages, lymphocytes, dendritic cells, cytokines, and chemokines, with reported effects in models of immunosuppression and tumor-associated immune dysfunction. Together, these two reviews illustrate the broad biological potential attributed to fungal polysaccharides while also identifying an important gap: much of the available evidence remains preclinical, and the relationship between molecular structure, preparation methods, bioavailability, microbiota metabolism, and biological activity requires further clarification [10,12].

The nutritional dimension of natural bioactive molecules is represented particularly well by the studies on mushrooms and food structure. Đogo Mračević et al. [13] evaluated ergosterol concentrations in wild-growing and cultivated edible mushrooms from Serbia and Korea using square-wave voltammetry. Ergosterol concentrations varied substantially among species and were generally higher in cultivated mushrooms and in mushroom stems compared to caps, with principal component analysis identifying a notable relationship between iron and ergosterol content. Beyond its nutritional relevance, ergosterol is of particular interest as a precursor to vitamin D2 and as a molecule with potential pharmacological properties [14]. Luo et al. [15] approached natural bioactive molecules from a different but highly translational perspective, investigating ultrasound-treated, psyllium husk-stabilized emulsion gels containing inulin for the development of foods that are suitable for individuals with oropharyngeal dysphagia. Increasing inulin concentration under ultrasound treatment markedly reduced droplet size, improved emulsion stability, increased matrix cohesion, and lowered expressible fluid content in purees, fulfilling the International Dysphagia Diet Standardisation Initiative (IDDSI) Level 4 requirements while demonstrating increased inhibition of α-amylase and α-glucosidase. Finally, Szendzielorz and Spiewak [16] reviewed the potential role of caffeine in cosmetic products targeting hair loss, detailing several mechanisms through which caffeine may influence hair follicle biology, including interactions with adenosine pathways, modulation of intracellular cAMP, metabolic effects, and antioxidant activity. Although the experimental and clinical findings are promising, the review appropriately underlined that much of the available clinical evidence is limited by methodological weaknesses, providing an important example of the distinction between biological plausibility and robust clinical evidence in commercial health or cosmetic applications.

Taken together, the nine contributions demonstrate the remarkable breadth of the field of bioactive and nutritional molecules. Despite this diversity, several common challenges emerge. Chemical characterization alone is insufficient to establish therapeutic relevance, meaning that bioactivity must ultimately be interpreted in relation to absorption, metabolism, tissue distribution, achievable concentrations, molecular targets, and safety [6,12]. Furthermore, complex natural extracts and mixtures remain difficult to standardize because their composition can vary according to species, cultivation or environmental conditions, extraction procedures, processing, and storage [17]. To overcome these limits, the substantial body of in vitro and animal evidence needs to be complemented by well-designed human studies that are capable of establishing clinically meaningful effects [18], while greater attention should be given to structure–activity relationships, bioavailability, metabolite formation, interactions with the gut microbiota, and potential interactions with conventional drugs [19]. Future research should therefore increasingly move toward integrated and translational approaches, combining advanced analytical chemistry with metabolomics, molecular biology, computational approaches, microbiome research, and appropriately designed clinical investigations. Such approaches may help to distinguish compounds with genuine therapeutic or nutritional potential from those whose effects remain limited to experimental models. Equally important will be the development of standardized preparations and robust biomarkers that are capable of linking exposure to biological response [18,20].
